# Half-body MRI volumetry of abdominal adipose tissue in patients with obesity

**DOI:** 10.1186/s12880-019-0383-8

**Published:** 2019-10-22

**Authors:** Nicolas Linder, Kilian Solty, Anna Hartmann, Tobias Eggebrecht, Matthias Blüher, Roland Stange, Harald Busse

**Affiliations:** 1 Department of Diagnostic and Interventional Radiology, Leipzig University Hospital, Leipzig, Germany; 2 Integrated Research and Treatment Center (IFB) AdiposityDiseases, Leipzig University Medical Center, Leipzig, Germany; 3 Department of Medicine, Endocrinology and Nephrology, Leipzig University, Leipzig, Germany

**Keywords:** Magnetic resonance imaging, Adipose tissue, Quantification, Segmentation, Volumetry, Obesity

## Abstract

**Background:**

The purpose of this study was to determine to what extent the whole volumes of abdominal subcutaneous (ASAT) and visceral adipose tissue (VAT) of patients with obesity can be predicted by using data of one body half only. Such a workaround has already been reported for dual-energy x-ray absorption (DEXA) scans and becomes feasible whenever the field of view of an imaging technique is not large enough.

**Methods:**

Full-body abdominal MRI data of 26 patients from an obesity treatment center (13 females and 13 males, BMI range 30.8–41.2 kg/m^2^, 32.6–61.5 years old) were used as reference (REF). MRI was performed with IRB approval on a clinical 1.5 T MRI (Achieva dStream, Philips Healthcare, Best, Netherlands). Segmentation of adipose tissue was performed with a custom-made Matlab software tool. Statistical measures of agreement were the coefficient of determination *R*^2^ of a linear fit.

**Results:**

Mean ASAT_REF_ was 12,976 (7812–24,161) cm^3^ and mean VAT_REF_ was 4068 (1137–7518) cm^3^. Mean half-body volumes relative to the whole-body values were 50.8% (48.2–53.7%) for ASAT_L_ and 49.2% (46.3–51.8%) for ASAT_R_. Corresponding volume fractions were 56.4% (51.4–65.9%) for VAT_L_ and 43.6% (34.1–48.6%) for VAT_R_. Correlations of ASAT_REF_ with ASAT_L_ as well as with ASAT_R_ were both excellent (*R*^2^ > 0.99, *p* < 0.01). Corresponding correlations of VAT_REF_ were marginally lower (*R*^2^ = 0.98 for VAT_L_, *p* < 0.01, and *R*^2^ = 0.97 for VAT_R_, *p* < 0.01).

**Conclusions:**

In conclusion, abdominal fat volumes can be reliably assessed by half-body MRI data, in particular the subcutaneous fat compartment.

## Background

The increasing worldwide prevalence of obesity poses serious health and economic problems [1]. Obesity is characterized by the abundance of ectopic adipose tissue, which can be divided into visceral and subcutaneous fat with specific metabolic functions [2]. Visceral obesity is generally considered to have a negative impact on health resulting in an increased risk for cardiometabolic diseases such as diabetes mellitus type 2 or atherosclerosis, whereas excess subcutaneous fat is still discussed controversially [3, 4]. Various clinical trials have already used magnetic resonance imaging (MRI) to non-invasively characterize obesity [5]. Visceral and other ectopic fat volumes are usually quantified by segmentation of multiplanar images derived from computed tomography or magnetic resonance imaging. Quantitative measures of body composition can be essential for the monitoring of therapeutic approaches of patients with obesity such as sport interventions [6], pharmacological trials [7] or bariatric surgery [8–11].

For larger patients, the imaging field of view (FOV) of an MRI system (typically 50–55 cm) may be too small to cover the whole body laterally. Moreover, field distortions, spatial inhomogeneities of the applied electromagnetic pulses and imaging artefacts at the edges of the FOV may preclude proper image analysis. Dual energy X-ray absorptiometry (DEXA) measurements are also subject to weight and scan area restrictions for patients with obesity [12].

Surrogate DEXA measurements of one body half only have already been proposed in the mid-1990s to overcome these limitations [12, 13]. Considering the approximate mirror symmetry of the human body (with respect to the median plane), we hypothesized that the total abdominal subcutaneous adipose tissue volume can be predicted by half-body data only. The goal of this work was to test this hypothesis for patients with obesity where the available MRI data still covers the entire lateral body.

## Methods

### Study population

MRI data at 1.5 T were available from a total of 224 patients (60 male) from an interventional clinical trial on obesity at a single institutional research center. Subjects with a BMI above 30 kg/m^2^ (inclusion criterion) underwent MRI as part of a clinical characterization for the local obesity biobank. No additional imaging was performed for this retrospective analysis. Thirty-six of the male patients (60%) were excluded because subcutaneous fat amounts on any of the abdominal MR images (slice thickness 10 mm) were not fully contained within the field of view or showed image artifacts that prevented precise segmentation. Another 11 male patients were excluded because the upper landmark for the segmentation of abdominal subcutaneous fat (vertrebra T9, see below) was not included in the trial dataset. The remaining 13 male patients were matched for age to 13 female patients. The mean BMI was 34.3 (range 30.8–41.2) kg/m^2^.

### Magnetic resonance imaging

Data were acquired on a standard clinical system that was upgraded from 1.5 to 3 Tesla throughout the original clinical trial (Achieva XR and dSTREAM, Philips, Best, Netherlands). For this analysis, however, we only considered one field strength (1.5 T) to reduce variability. Patients were examined in supine position with arms on the side and images were acquired in breath-hold technique (expiration) using the whole-body coil for signal reception. Fat-sensitive transverse MR images (two-point Dixon sequence, slice thickness 10 mm, interslice gap 0.5 mm) were acquired to minimally include the abdominal region between diaphragm and pelvic floor using two contiguous stacks of 25 images each. Our measurement of abdominal subcutaneous adipose tissue (ASAT) volume, however, relied on a fixed landmark (vertebra T9) rather the more variable position of the diaphragm as recommended by Ulrich et al. [14]. Further technical details, including all relevant MR parameters, can be found in a previous report [15, 16].

### Image analysis

**Fig. 1:**
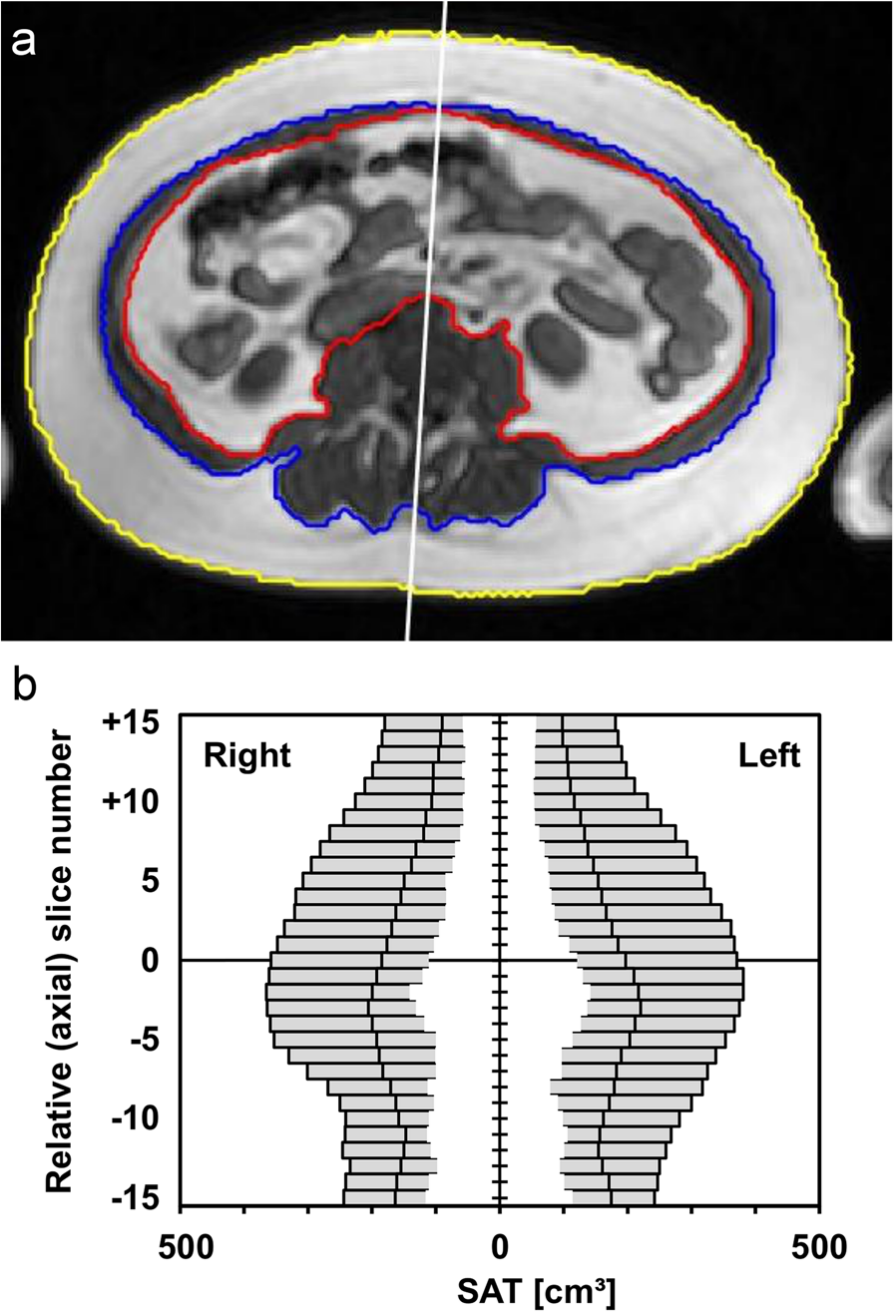
Quantification of abdominal adipose tissue in MRI. **a** Screenshot of the segmentation software (Matlab). The manually drawn median line is meant to separate the two body halves. Colored lines mark the outer (yellow) and inner (blue) ASAT boundaries and a contour (red) encompassing the VAT components. The tool is available from an online repository (https://github.com/Stangeroll/Dicomflex). **b** Distribution of partial ASAT volumes for left and right body halves as a function of relative (axial) slice number for all subjects (slice spacing: 10.5 mm). Outer, middle and inner vertical marks represent maximum, median and minimum values. Slice position 0 corresponds to the level of the umbilicus

A custom-made software tool was used to semi-automatically segment the half-body adipose tissue areas after proper marking of the median line. This tool was developed under the Matlab-based Dicomflex framework [17] and is available in the Github software repository (https://github.com/Stangeroll/Dicomflex). Validation against a reference software was reported earlier [18]. The abdominal adipose tissue areas were identified by a trained experienced reader (A.H.) on all transverse slices (see above). Figure 1 shows an example of such a segmentation.

The fully segmented abdominal subcutaneous and visceral adipose tissue served as reference standard (ASAT_REF_ and VAT_REF_). At the level of lumbar vertebra 4 or 5 between the dorsal aspect of the processus spinosus and the center of the corresponding vertebra, a reference median line dividing total ASAT into proper left and right portions (ASAT_L_ and ASAT_R_) was drawn manually. This line was digitally pasted into all slices but could be modified in each slice to correct for potential scoliotic deformations.

### Statistical analysis

Left and right half-body volumes were then plotted against the reference volumes. A linear fit yielded specific slopes and intercepts that can be regarded as conversion parameters between half and full measures:

1 $$ {\mathrm{ASAT}}_{\mathrm{EST}-\left[\mathrm{L}/\mathrm{R}\right]}={\mathrm{ASAT}}_{\left[\mathrm{L}/\mathrm{R}\right]}\cdot 1/{f}_{\mathrm{ASAT}-\left[\mathrm{L}/\mathrm{R}\right]}+{b}_{\mathrm{ASAT}-\left[\mathrm{L}/\mathrm{R}\right]} $$

2 $$ {\mathrm{VAT}}_{\mathrm{EST}-\left[\mathrm{L}/\mathrm{R}\right]}={\mathrm{VAT}}_{\left[\mathrm{L}/\mathrm{R}\right]}\cdot 1/{f}_{\mathrm{VAT}-\left[\mathrm{L}/\mathrm{R}\right]}+{b}_{\mathrm{VAT}-\left[\mathrm{L}/\mathrm{R}\right]} $$

where the index [L/R] denotes either the left or the right body side, ASAT_EST-[L/R]_ and VAT_EST-[L/R]_ are the estimated total fat volumes, ASAT_[L/R]_ and VAT_[L/R]_ are the partially measured volumes and ***f***_**ASAT-[L/R]**_ and ***b***_**ASAT-[L/R]**_ are the slope [no unit] and intercept [unit of volume] parameters of the corresponding linear fits.

Statistical measures of agreement were the coefficient of determination *R*^2^ of a linear fit, and Bland-Altman analyses between measured and predicted values. A Shapiro-Wilk statistic was considered to test for a normal distribution of the respective differences. A two-sided T-test was used to compare both genders in regards to BMI and age. All statistical analyses were performed with SPSS 24 (IBM, Armonk, NY) and *p*-values below 0.05 were considered to be significant.

## Results

**Fig. 2:**
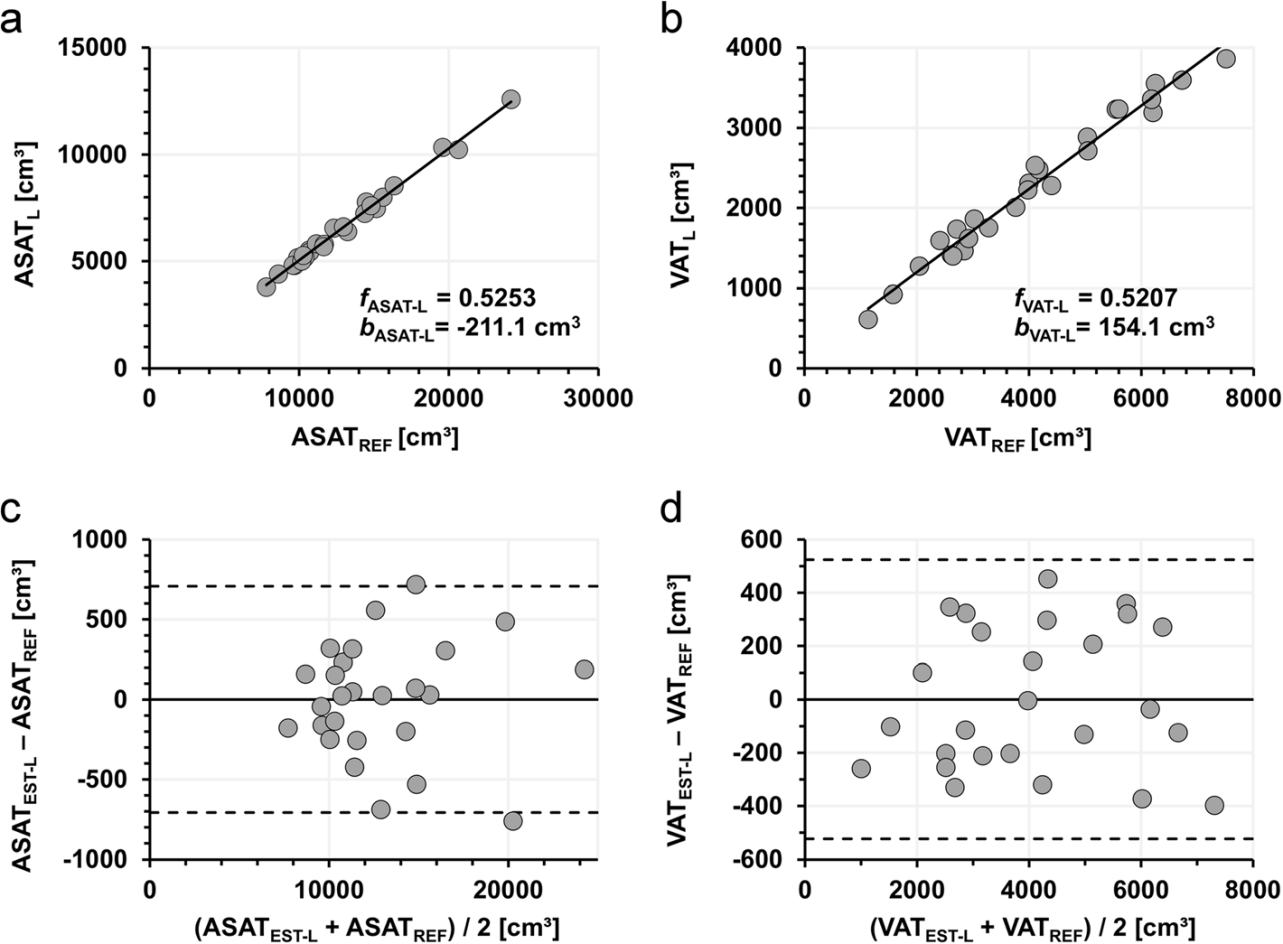
Correlation of half-body and full-body ASAT and VAT measurements. Linear fits through the data (**a** and **b**) are represented by solid lines. Coefficients of determination were *R*^2^ = 0.99 for ASAT (**a**) and *R*^2^ = 0.98 for VAT (**b**). Corresponding Bland-Altman plots for ASAT (**c**) and VAT (**d**) reveal good agreement between both methods

Data of 13 female and 13 male individuals were included. Mean BMI was 34.3 (range 30.8–41.2) kg/m^2^ and mean age was 50.0 (range 32.6–61.5) years. Gender-specific patient characteristics are provided in Table 1. There was no statistical difference in age (*p* = 0.571) or BMI (*p* = 0.525) between genders. Image segmentation and determination of VAT_REF_, VAT_L_, VAT_R_, ASAT_REF_, ASAT_L_ and ASAT_R_ could be successfully performed for all patients. Definition of the median line took about 2 min and total segmentation time was about 12 min per patient. Mean volumes of abdominal subcutaneous (ASAT_REF_) and visceral adipose tissue (VAT_REF_) were 12,976 (range 7812 – 24,161) cm^3^ and 4068 (1137 – 7518) cm^3^, respectively. Mean volumes of ASAT_L_ and ASAT_R_ were 6605 (3799 – 12,579) cm^3^ and 6370 (4013–11,582) cm ^3^. Mean volumes of VAT_L_ and VAT_R_ were 2272 (611–3859) cm^3^ and 1795 (526–3654) cm^3^. Figure 2 illustrates the linear correlation between ASAT_L_ and ASAT_REF_. Coefficients of determination were *R*^2^ > 0.99 over all patients. Values of ASAT_EST-L_ were significantly higher in females compared to males (15,020 vs. 10,932 cm^3^). Coefficients *R*^2^ between either ASAT_L_ or ASAT_R_ with ASAT_REF_ were very high (0.99) and did not differ significantly between genders. In contrast, correlations between ASAT_L_ and BMI were poor for both females (*R*^2^ = 0.26, *p* < 0.01) and males (*R*^2^ = 0.35, p < 0.01).

**Table 1:** Patient characteristics

	Females	Males
Count	13	13
Age [years]	49.0 (3.9–61.0)	50.9 (32.6–61.5)
BMI [kg/m^2^]	34.9 (31.4–37.3)	33.7 (30.8–41.2)
ASAT_REF_ [cm^3^]	15,020 (10,672 – 24,161)	10,932 (7812 –16,349)
VAT_REF_ [cm^3^]	2786 (1137 – 4174)	5350 (3282 –7513)

Data of age and BMI are presented as mean and corresponding range

Presented *p* values are derived from a t-Test on equality of variances

*BMI* body mass index, *ASAT*_*REF*_ reference abdominal subcutaneous adipose tissue (volume), *VAT*_*REF*_ reference visceral adipose tissue (volume)

Considering VAT, females had a significantly (*p* < 0.01) lower mean volume (2787 cm^3^) than males (5350 cm^3^). Coefficients of determination between VAT_L_ or VAT_R_ with VAT_REF_ were both very good (*R*^2^ = 0.98 and 0.97, respectively, both *p* < 0.01). For VAT_R_, *R*^2^ was slightly better for males (*R*^2^ = 0.95) than for females (*R*^2^ = 0.90). Correlation with BMI was moderate in males (*R*^2^ = 0.46) and practically not given in females (*R*^2^ = 0.05).

Conversion parameter sets were {*f*_ASAT-L_ *=* 0.5253, *b*_ASAT-L_ = − 211.1 cm^3^}, {*f*_ASAT-R_ = 0.4747, *b*_ASAT-R_ = 211.1 cm^3^}, {*f*_VAT-L_ = 0.5207, *b*_VAT-L_ = 154.1 cm^3^} and {*f*_VAT-R_ = 0.4793, *b*_VAT-R_ = -154.1 cm^3^}. Mean values of the derived estimates were VAT_EST-L_ = 4069.2, VAT_EST-R_ = 4068.4, ASAT_EST-L_ = 12,976.4 and ASAT_EST-R_ 12,976,2. As a prerequisite for Bland-Altman analysis, the null hypothesis of volume differences coming from a normally distributed population could not be rejected (*p*-values between 0.051 and 0.931). The Bland-Altman plots for the left side (Fig. 2c and d) reveal a balanced distribution over the whole range of fat values with standard deviations of 361 cm^3^ and 267 cm^3^ for ASAT and VAT, respectively.

## Discussion

**Fig. 3:**
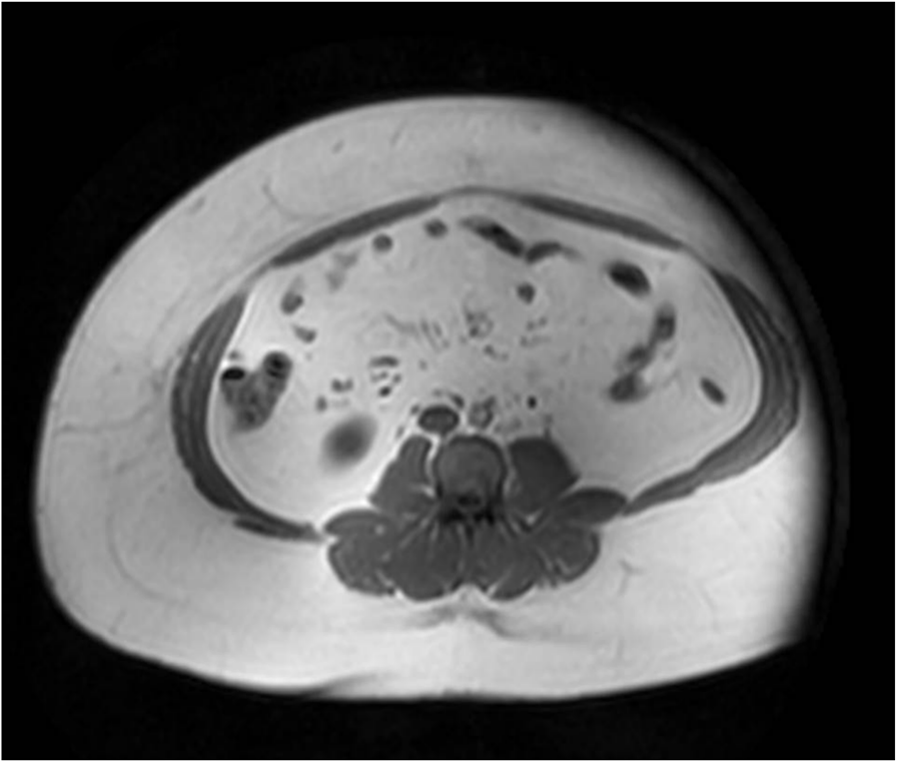
Suggested workaround for adipose tissue quantification in patients with higher degrees of obesity. Sample transverse MR image after patient has been positioned non-centrally (laterally) on the MR table. Full-body fat amounts can be estimated from half-body measures (here: right) using reference/ conversion parameters derived here. MRI acquisition with (obese) patient in central (normal) position is prone to image artifacts or (anatomical) cutoffs on both sides which would prevent proper prediction

Quantification of abdominal subcutaneous adipose tissue (ASAT) in patients with obesity is typically compromised by imaging limitations. Earlier reports of partial coverage of abdominal adipose tissue focused on either single slice or partial volume quantification and where mainly concentrating on visceral adipose tissue [15, 16, 19–21]. Therefore, the main objective of this study was to implement and evaluate a technique that estimates the ASAT volume of a patient from half-body data only. Here, validation was only performed for MRI datasets where the lateral body parts were fully contained in the FOV. Larger patients, in which these parts would normally be cut off, could then be placed with a lateral offset on the MRI table (see Fig. 3) to fully include one body half instead, preferentially the left one.

Our results revealed an excellent correlation between ASAT_REF_ volumes and estimates from ASAT_L_ or ASAT_R_ with a slightly better agreement on the left side. This finding agrees with results from dual-energy X-ray absorptiometry [12] and also supports the assumption of a nearly symmetric ASAT distribution. Despite the pronounced lateral asymmetry of abdominal organs like the liver or spleen, VAT may still be predicted by half-body data. This may be explained by the observation that VAT is predominantly found in the lower two thirds of the abdomen where intestinal and pelvic structures show no distinct lateral preference. VAT volumes next to the liver and spleen are rather asymmetric but make up a small amount of total VAT only. In males, VAT_R_ should be preferred for VAT prediction; in females, differences between VAT_L_ and VAT_R_ were only marginal.

Our pilot study has some limitations. Like in other studies involving MRI segmentation of adipose tissue areas [18, 22], our sample size is relative small. Although the original trial data included patients with a maximum BMI of 57 kg/m^2^, the strict inclusion criteria applied for validation here (all ASAT boundaries within FOV, no artifacts, available MRI data at position T9) resulted in an effective BMI range of 30–41 kg/m^2^ only. The good agreement may therefore not hold for subjects with higher degrees of obesity. Our semi-automatic segmentation tool has been used for all clinical analyses as well and requires more processing time than the latest fully-automated approaches [23, 24]. Data were deliberately analyzed by one operator only to exclude variations during interactive segmentation and median-line definition. Results of our retrospective analysis were not validated against an independent method. Also, DEXA scans had been excluded from the clinical study protocol to avoid application of ionizing radiation. Ultrasound was not considered either because the underlying accuracy is also low [25]. Despite the limited availability and higher complexity, MRI is used increasingly and even referred to as a gold standard for the quantification of adipose tissue. Furthermore, the presented results should be transferable to computed tomography, which comprises an almost identical imaging geometry.

## Conclusion

In conclusion, we have presented a unique workaround method to reliably quantify abdominal adipose tissue in patients with higher grades of obesity using MRI. It is of particular value for ASAT but may also be used to estimate VAT with slightly lower accuracy. We believe that this simple half-body MRI volumetry has a high practical value for characterization of obesity, both in research and treatment.

Future work should be directed towards an independent validation, a more standardized image segmentation and a potential definition of normative values like the ones recently reported for a normal-weight Swiss population [14]. Our Matlab tool, the source code and the corresponding framework are therefore available from a Github repository (https://github.com/Stangeroll/Dicomflex) to facilitate further efforts along that line [17].

## Data Availability

Data is available upon request from the corresponding author (nicolas.linder@medizin.uni-leipzig.de).

## Abbreviations

ASAT_EST-L_: Abdominal subcutaneous adipose tissue estimated from the left side of the body
ASAT_EST-R_: Abdominal subcutaneous adipose tissue estimated from the right side of the body
ASAT_L_: Abdominal subcutaneous adipose tissue on the left side of the body
ASAT_R_: Abdominal subcutaneous adipose tissue on the right side of the body
ASAT_REF_: Abdominal subcutaneous adipose tissue on both sides of the body (reference)
BMI: Body mass index
FOV: Field of view
MRI: Magnetic resonance imaging
VAT_L_: Visceral adipose tissue on the left side of the body
VAT_R_: Visceral adipose tissue on the right side of the body
VAT_REF_: Visceral adipose tissue on both sides of the body (reference)

## References

[1] NCD Risk Factor Collaboration (NCD-RisC). Worldwide trends in body-mass index, underweight, overweight, and obesity from 1975 to 2016: a pooled analysis of 2416 population-based measurement studies in 128·9 million children, adolescents, and adults. Lancet. 2017;390(10113):2627–42. 10.1016/S0140-6736(17)32129-3 PMC5735219 29029897

[2] Sam S. Differential effect of subcutaneous abdominal and visceral adipose tissue on cardiometabolic risk. Horm Mol Biol Clin Invest. 2018;33(1). 10.1515/hmbci-2018-0014 29522417

[3] Merlotti C, Ceriani V, Morabito A, Pontiroli AE (2017). Subcutaneous fat loss is greater than visceral fat loss with diet and exercise, weight-loss promoting drugs and bariatric surgery: a critical review and meta-analysis. Int J Obes.

[4] Wajchenberg B. L., Giannella-Neto D., da Silva M. E., Santos R. F. (2002). Depot-Specific Hormonal Characteristics of Subcutaneous and Visceral Adipose Tissue and their Relation to the Metabolic Syndrome. Hormone and Metabolic Research.

[5] Lemos T, Gallagher D (2017). Current body composition measurement techniques. Curr Opin Endocrinol Diabetes Obes.

[6] Mueller SM, Anliker E, Knechtle P, Knechtle B, Toigo M (2013). Changes in body composition in triathletes during an ironman race. Eur J Appl Physiol.

[7] Middleton MS, Heba ER, Hooker CA, Bashir MR, Fowler KJ, Sandrasegaran K (2017). Agreement between magnetic resonance imaging proton density fat fraction measurements and pathologist-assigned steatosis grades of liver biopsies from adults with nonalcoholic steatohepatitis. Gastroenterology..

[8] Toro-Ramos Tatiana, Goodpaster Bret H., Janumala Isaiah, Lin Susan, Strain Gladys W., Thornton John C., Kang Patrick, Courcoulas Anita P., Pomp Alfons, Gallagher Dympna (2014). Continued loss in visceral and intermuscular adipose tissue in weight-stable women following bariatric surgery. Obesity.

[9] Meyer-Gerspach AC, Peterli R, Moor M, Madörin P, Schötzau A, Nabers D (2019). Quantification of liver, subcutaneous, and visceral adipose tissues by mri before and after bariatric surgery. Obes Surg.

[10] Hui SCN, Wong SKH, Ai Q, Yeung DKW, Ng EKW, Chu WCW (2019). Observed changes in brown, white, hepatic and pancreatic fat after bariatric surgery: evaluation with MRI. Eur Radiol.

[11] Luo RB, Suzuki T, Hooker JC, Covarrubias Y, Schlein A, Liu S (2018). How bariatric surgery affects liver volume and fat density in NAFLD patients. Surg Endosc.

[12] Rothney MP, Brychta RJ, Schaefer EV, Chen KY, Skarulis MC. Body composition measured by dual-energy X-ray absorptiometry half-body scans in obese adults. Obesity (Silver Spring). 2009;17(6):1281–6. 10.1038/oby.2009.14 PMC2709755 19584885

[13] Tataranni PA, Ravussin E (1995). Use of dual-energy X-ray absorptiometry in obese individuals. Am J Clin Nutr.

[14] Ulbrich EJ, Nanz D, Leinhard OD, Marcon M, Fischer MA (2018). Whole-body adipose tissue and lean muscle volumes and their distribution across gender and age: MR-derived normative values in a normal-weight Swiss population. Magn Reson Med.

[15] Schaudinn A, Linder N, Garnov N, Kerlikowsky F, Blüher M, Dietrich A (2015). Predictive accuracy of single- and multi-slice MRI for the estimation of total visceral adipose tissue in overweight to severely obese patients: MRI prediction of visceral fat volumes. NMR Biomed.

[16] Linder N, Schaudinn A, Garnov N, Blüher M, Dietrich A, Schütz T, et al. Age and gender specific estimation of visceral adipose tissue amounts from radiological images in morbidly obese patients. Sci Rep. 2016;6:22261. 10.1038/srep22261 PMC4806365 27009353

[17] Stange R, Linder N, Schaudinn A, Kahn T, Busse H (2018). Dicomflex: a novel framework for efficient deployment of image analysis tools in radiological research. PLoS One.

[18] Thörmer G, Bertram HH, Garnov N, Peter V, Schütz T, Shang E (2013). Software for automated MRI-based quantification of abdominal fat and preliminary evaluation in morbidly obese patients. J Magn Reson Imaging.

[19] Irlbeck T, Massaro JM, Bamberg F, O’Donnell CJ, Hoffmann U, Fox CS (2010). Association between single-slice measurements of visceral and abdominal subcutaneous adipose tissue with volumetric measurements: the Framingham heart study. Int J Obes.

[20] Marzetti M, Brunton T, McCreight L, Pearson E, Docherty S, Gandy SJ (2018). Quantitative MRI evaluation of whole abdomen adipose tissue volumes in healthy volunteers-validation of technique and implications for clinical studies. Br J Radiol.

[21] Schwenzer Nina F., Machann Jürgen, Schraml Christina, Springer Fabian, Ludescher Burkhard, Stefan Norbert, Häring Hans, Fritsche Andreas, Claussen Claus D., Schick Fritz (2010). Quantitative Analysis of Adipose Tissue in Single Transverse Slices for Estimation of Volumes of Relevant Fat Tissue Compartments. Investigative Radiology.

[22] Zhou A, Murillo H, Peng Q (2011). Novel segmentation method for abdominal fat quantification by MRI. J Magn Reson Imaging.

[23] Borga M, Thomas EL, Romu T, Rosander J, Fitzpatrick J, Dahlqvist Leinhard O (2015). Validation of a fast method for quantification of intra-abdominal and subcutaneous adipose tissue for large-scale human studies: quantification of IAAT and ASAT. NMR Biomed.

[24] Langner Taro, Hedström Anders, Mörwald Katharina, Weghuber Daniel, Forslund Anders, Bergsten Peter, Ahlström Håkan, Kullberg Joel (2018). Fully convolutional networks for automated segmentation of abdominal adipose tissue depots in multicenter water–fat MRI. Magnetic Resonance in Medicine.

[25] Murphy J, Bacon SL, Morais JA, Tsoukas MA, Santosa S. Intra-abdominal adipose tissue quantification by alternative versus reference methods: a systematic review and meta-analysis. Obesity (Silver Spring). 2019;27(7):1115–22. 10.1002/oby.22494 31131996

